# Use of an automatic fruit-zone cooling system to cope with multiple summer stresses in Sangiovese and Montepulciano grapes

**DOI:** 10.3389/fpls.2024.1391963

**Published:** 2024-04-10

**Authors:** Gabriele Valentini, Gianluca Allegro, Chiara Pastore, Daniela Sangiorgio, Massimo Noferini, Enrico Muzzi, Ilaria Filippetti

**Affiliations:** ^1^ Department of Agricultural and Food Sciences, University of Bologna, Bologna, Italy; ^2^ iFarming srl, Ravenna, Italy

**Keywords:** anthocyanin, climate change, phenolic maturity, precision irrigation, viticulture, water stress

## Abstract

Grapevines are frequently subjected to heatwaves and limited water availability during ripening. These conditions can have consequences for the physiological health of the vines. Moreover, the situation is often exacerbated by intense solar radiation, resulting in reduced yield due to sunburn and a decline in quality. In light of these challenges, our study aimed to develop a fruit-zone cooling system designed to mitigate grape sunburn damage and improve the microclimate conditions within the vineyard. The system comprises a network of proximal sensors that collect microclimate data from the vineyard and an actuator that activates nebulizers when the temperature exceeds the threshold of 35°C. The research was conducted over two years (2022 and 2023) in Bologna (Italy) using potted Sangiovese and Montepulciano vines. These two vintages were characterized by high temperatures, with varying amounts of rainfall during the test period, significantly impacting the evaporative demand, which was notably higher in 2023. Starting from the veraison stage we compared three treatments: Irrigated control vines (WW); Control vines subjected to 50% water restriction during the month of August (WS); WS vines treated with nebulized water in the bunch area during the stress period (WS+FOG). The application of nebulized water effectively reduced the temperature of both the air around the clusters and the clusters themselves. As we expected, Montepulciano showed better single leaf assimilation rate and stomatal conductance under non-limiting water conditions than Sangiovese while their behavior was unaffected under water-scarce conditions. Importantly, for the first time, we demonstrated that nebulized water positively affected gas exchange in both grape varieties. In addition to this, the vines treated with the misting system exhibited higher productivity compared to WS vines without affecting technological maturity. In the 2023 vintage, the activation of the system prevented the ripening blockage that occurred in Montepulciano under water stress. Regarding the concentration of total anthocyanins, a significant increase in color was observed in WS+FOG treatment, suggesting a predominant role of microclimate on anthocyanin biosynthesis and reduction of oxidative phenomena. In conclusion, the fruit-zone cooling system proved to be an invaluable tool for mitigating the adverse effects of multiple summer stresses.

## 1 Introduction

Sangiovese and Montepulciano (*Vitis vinifera* L.) are the main red varieties grown in Italy (IOVW, 2017). Sangiovese is grown in central and northern Italy and reached 50 million hectoliters of production in 2022 (IOVW, 2022). Montepulciano, on the other hand, is a variety grown in central-southern Italy that becomes part of many red and rosé wines with appellations of origin such as Montepulciano d’Abruzzo and Cerasuolo d’Abruzzo. In the past few decades, Italian vineyards have faced heightened challenges due to pronounced exposure to intense solar radiation, elevated vapor pressure deficits (VPDs) and soaring temperatures, particularly during the crucial ripening phase in post-veraison (Jones et al., 2005; Mariani et al., 2009; Tomasi et al., 2011; Di Carlo et al., 2019). Consequently, indigenous grape varieties are experiencing increasingly harsh conditions during their reproductive stage, leading to a spatial redistribution phenomenon. This has resulted in a notable geographical shift, with areas conducive to viticulture moving towards cooler regions (Lereboullet et al., 2014; van Leeuwen et al., 2020). A notable case in point is observed in Abruzzo region (Italy) where the historical harvest period for Trebbiano abruzzese extended from the second week of October during the 1800s and 1900s (Di Carlo et al., 2019). In contemporary times, however, the harvest of this variety begins in the second week of September, a strategic adjustment aimed at mitigating the adverse effects of heat on berry ripening. This alteration poses a potential risk to the quality of the grapes, as they tend to accumulate higher sugar content, potentially compromising their aromatic qualities (Palliotti et al., 2014).

According to several authors, *Vitis vinifera* L. exhibits a general anisohydric behavior in conditions of water scarcity and thermo-radiative excess (Tombesi et al., 2016; Valentini et al., 2022). In greater detail, the mentioned grape varieties showcase distinct responses to drought conditions, particularly in terms of stomatal control: Montepulciano (near-isohydric) demonstrates nearly complete stomatal closure at leaf potentials less negative than those observed in Sangiovese, which is considered anisohydric (Dal Santo et al., 2016). This mechanism aims to prevent excessive tissue dehydration and protects the plant vessels from the risk of hydraulic failure (Tyree and Sperry, 1988). Consequently, this adaptation to environmental stimuli primarily involves stomatal behavior to limit water loss while optimizing CO_2_ assimilation (Schultz, 1996; Bota et al., 2001). Nevertheless, under heightened stress conditions, vines often exhibit leaves with chlorosis and necrosis, leading to a significant reduction in marketable yield due to sunburn (Dinis et al., 2016). This condition results in grapes with a low organic acid content, high pH and poor color due to anthocyanin degradation (Allegro et al., 2020).

To reduce these adverse climate effects, some short-term agronomic techniques are now available, such as foliar application of kaolin and zeolite (Calzarano et al., 2019; Valentini et al., 2021) and smart irrigation (Costa et al., 2016; Fraga et al., 2018). The mist-irrigation application was extensively studied in the 1970s, when it was promoted as an efficient method for reducing plant water stress (Howell et al., 1971). In particular, the use of overhead sprinklers in various cultivations triggered satisfactory results in heat reduction (Chesness and Braud, 1970; Gilbert et al., 1970; Robinson, 1970). Therefore, an ultra-fine misting system was developed to induce evaporative cooling in grapevine canopies, reducing both water consumption and leaf wetting, and in turn plant diseases (Matthias and Coates, 1986). Interesting results on thermal control were obtained on Cabernet Sauvignon with an evaporative cooling system placed inside the canopy activated manually based on weather forecasts (Caravia et al., 2017) and in Semillon by the Hydrocooling system (Greer and Weedon, 2014).

Starting from these assumptions, a new fruit-zone cooling system was implemented in the experimental vineyard of the University of Bologna, in which multiple stresses often occur in summer, to improve the microclimate conditions of the grapes during the ripening. The fully automated system was tested on potted Sangiovese and Montepulciano varieties to verify the effects of misting on gas-exchanges, yield and berry composition.

## 2 Materials and methods

### 2.1 Plant material and experimental design

The trials were carried out over the 2022 and 2023 seasons on potted Sangiovese and Montepulciano vines at the experimental station of the University of Bologna (Bologna, 44°32’N, 11°22’E). Plant material consisted of Sangiovese (clone TEA 10D) and Montepulciano (clone R7) vines, both grafted onto 110R rootstock, spaced at 1 m within the row and 2.5 m between rows. The vines were oriented northeast to southwest and trained to a vertical shoot positioned spur-pruned cordon. Winter pruning left 10 buds while during spring 10 shoots per vine were left. The pot experimental design included 30 vines uniformed in cluster number and randomly distributed (RCD). The vines were planted in 2019 on 30 L pots filled with a soil mixture (39% sand, 39% silt and 22% clay) with an organic matter content of 1.8% and pH of 7.8. Field capacity and wilting point were calculated after Saxton and Willey (2005) and set at 0.29 cm^3^/cm^3^ and 0.14 cm^3^/cm^3^, respectively. The pots were kept well-watered until veraison, which occurred on the day of the year (DOY) 214 in 2022 and DOY 213 in 2023, providing the amount of water lost through transpiration – approximately 4 L day^-1^ of water – distributed automatically through a dripper irrigation system. From this date and throughout berry ripening, the vines underwent different irrigation treatment depending on the following treatments: well-watered vines that received 100% of the water lost by transpiration (WW); vines that received 50% of the water lost by transpiration (WS) until DOY 236 in 2022 and DOY 235 in 2023 respectively; vines that received 50% of the water lost by transpiration as WS and subjected to misting irrigation in the cluster zone (WS+FOG). In addition, each pot was entirely covered with both aluminum foil to avoid overheating and a plastic cover to avoid receiving rainwater and limiting soil evaporation. Each vine was fertilized with 10 g NPK Nitrophoska Gold (15-9-15) and the viticultural practices typical for the Emilia Romagna region were applied.

### 2.2 Fruit-zone cooling system characteristics

The fruit-zone cooling system was composed of both a wireless sensor network (WSN) able to acquire the microclimate data within the canopy and an actuator that triggers the nebulizers when the air temperature exceeds the threshold of 35°C. Additionally, the system could also be programmed to activate misting according to a Vapor Pressure Deficit (VPD) threshold calculated in real-time at the cluster zone thanks to its hardware part and the dedicated software. Furthermore, the system is composed of a hardware part: (a) a control unit powered by batteries recharged by a solar panel and connected to a network of sensors capable of continuously recording the relative humidity and temperature values around the fruit-zone (iFarming srl, Ravenna, RA, Italy); (b) a pipeline equipped with nebulizers which, at the operating pressure of 3.5 Bar, deliver drops of 50–55 microns in diameter. Each vine was equipped with a fogger (mod. RIVULIS F.L.F., Rivulis Irrigation, Gvat, Israel) with a flow rate of 10.4 L h^-1^. The fruit-zone cooling system has been located under bunch area (10 cm under the cordon) to limit the direct contact between water and leaves. The software part analyzed the microclimate data collected by the nodes, and sent the impulses to a solenoid valve that automatically regulated the opening and closing of the misting system. The air temperature of 35°C was chosen for turning on the system. Water was applied with the following cycle: on for 5 minutes, off for 15 minutes. At the end of each cycle, the system performed another air temperature check. Since the spacing both along and between rows was 1.0 m x 2.5 m, each vine received approximately 1 mm of water per hour (equivalent to 15 minutes of water spray).

### 2.3 Weather data acquisition

The weather conditions were recorded by a meteorological station annexed to the experimental vineyard (iFarming Srl, Ravenna, RA, IT). However, for the microclimate detail, six digital thermal probes and relative humidity sensors (iFarming, Ravenna, RA, Italy) were placed in the fruit zone within the canopy of the control and sprayed vines to detect the effect of misting on air temperature. Each sensor was then connected to a control unit to analyze in real-time, every 10 minutes, the microclimate data recorded by the sensor. Only for the year 2023, six thermocouples were inserted under the skin of WS and WS+FOG berries to continuously evaluate the effect of misting on fruit temperature. The thermocouples (iFarming srl, Ravenna, Italy) were inserted on berries of clusters distributed east, west and inside the canopy.

### 2.4 Physiological measurements

The leaf stomatal conductance (gs), the net photosynthesis (Pn), the intrinsic water-use efficiency (WUEi=Pn/gs) and the substomatal CO_2_ concentration (Ci) were evaluated using a portable gas exchange Li-Cor 6400 system (Li-Cor Inc., Lincoln, NE, USA) on three well-exposed main leaves per vine inserted between nodes 6 and 10. Leaf gas-exchange measurements were taken around midday on DOY 222 and 235 during 2022 year. Gas-exchanges were also assessed on DOY 219 and 223 during the 2023 season. These measurements were made with the precaution of shutting down the misting system for 45 minutes. On the same dates, the mean berry temperature (Tberry) was measured using an infrared thermometer (mod. Raynger ST, Raytek, Santa Cruz, CA, USA). Readings were taken on exposed and dry clusters (10 recordings per vine, 50 per treatment). At midday, the vine water status was evaluated by measuring the stem water potential (Ψstem) using a Scholander pressure chamber (Soilmoisture Corp., Santa Barbara, CA, USA). For each variety and dates a total of 15 leaves (five vines, one leaf per vine) per treatment were measured between 13:00 and 14:00 hours. Measurements were taken on leaves covered with aluminum foil and enclosed in a plastic bag for 90 minutes before reading.

### 2.5 Yield components and berry composition

At harvest, the clusters of tagged vines were counted and weighed to obtain yield. Moreover, 20 berries per vine (100 berries per treatment, 300 per each variety) were collected to analyze the following variables: total soluble solids concentration (TSS), using a self-compensating Maselli R50 refractometer (Misure Maselli, Parma, Italy); must pH and titratable acidity (TA), using a Crison titrator (Crison Instruments, Barcelona, Spain). At the same time, 20 berries for each vine were collected to evaluate total anthocyanins as reported by Mattivi et al. (2006). Anthocyanin analysis were performed with a Waters 1525 HPLC (Waters, Milford, MA) equipped with a diode array detector and a Phenomenex reversed-phase column with pre-column (Phenomenex, Castel Maggiore, Italy). Anthocyanins were quantified at 520 nm using an external calibration curve with malvidin-3-glucoside chloride as the standard (Sigma-Aldric, St. Louis, MO, USA). Sampling days were different for the two varieties under comparison: DOY 252 for Montepulciano and DOY 222, 234, 249 for Sangiovese in 2022; DOY 250 for both varieties in 2023. During winter the wood pruned from each vine was weighed (data were not reported because no difference was detected).

### 2.6 Statistical analysis

Data were processed for each variety by the analysis of variance over year using the mixed procedure of SAS v 9.0 (SAS Institute, Inc., Cary, NC, USA) and the treatment comparisons were analyzed using Tukey test with a cut-off at P ≤ 0.05.

## 3 Results

### 3.1 Weather conditions

**Figure 1:**
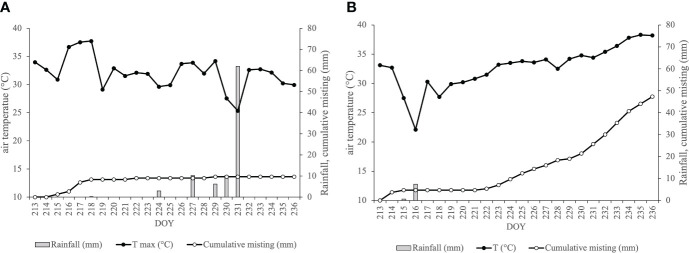
The cumulative misting (grey line, mm), the trend of air maximum temperature (black line, °C) and the rainfall (grey bars, mm) at a meteorological station during August (2022, **A**; 2023, **B**).

The 2022 season was characterized by warm and dry conditions, with a total of 260 mm of rainfall recorded from April to October. The weather data recorded during the entire trial period showed persistent heatwave during the first week of August, with air temperatures exceeding 35°C. In contrast, the third week of the month witnessed substantial rainfall as reported in **Figure 1**. Specifically, the experimental station documented 101 mm of rain during the stress period, as illustrated in **Figure 1**. In contrast, the 2023 season exhibited a different pattern, receiving 55 percent more rainfall than 2022, totaling about 560 mm during the vegetative months from April to October. Analyzing the thermo-pluviometric trend during the trial period, the first half of August was notably more humid, followed by severe heatwaves in the latter part of the month, where temperatures exceeded the established thermal threshold of 35°C (**Figure 1**). Unlike the 2022 vintage, 2023 witnessed reduced rainfall, accounting for only 10% of the previous year’s precipitation during the test period (10 mm versus 101.2 mm in 2023 and 2022, respectively). Notably, the difference between the seasons was the higher monthly relative humidity in the 2022 vintage, which had an impact on the vapor pressure deficit (VPD) throughout the entire test period.

Differences in 2022 and 2023 environmental conditions generated changes in system activation levels between the two observational periods. Specifically, during the 2022 season (**Figure 1A**), the cumulative water distribution reached an approximate value of 10 mm by the end of the trial period. This accumulation resulted from the misting system being activated on seven specific days at the beginning of August. This activation pattern was prompted by elevated air temperatures and a scarcity of rainfall events during this period. Conversely, the 2023 season exhibited a substantial increase in the cumulative water distributed, approximately 45 mm, concentrated in the latter part of the trial period spanning from DOY 222 to DOY 236 (**Figure 1B**). This is due to the higher frequency of system activations occurred in 2023, 15 vs the 7 days of 2022.

### 3.2 Microclimatic and physiological response

**Figure 2:**
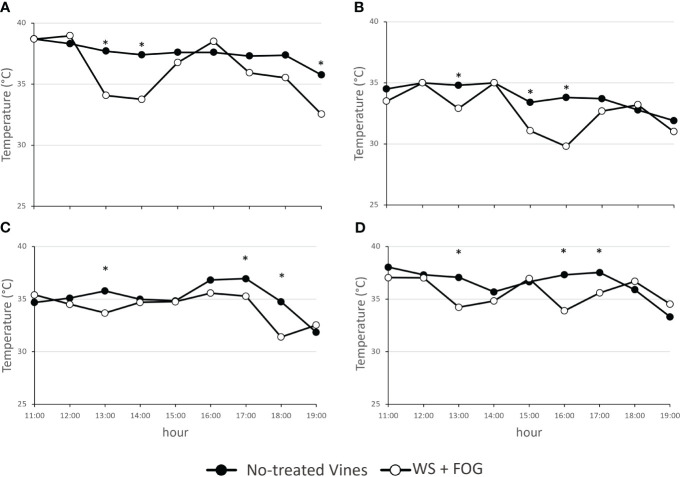
The trend of air temperature within the canopy on the fruit-zone during the warmest part of the day, from 11:00 to 19:00 hours, in untreated WW and WS vines (no-treated vines) and in vines treated with misting water (WS+FOG) in two representative days of 2022 (**A**, DOY 216; **B**, DOY 222) and of 2023 (**C**, DOY 228; **D**, DOY 233) seasons. Asterisks indicate significant differences between the two treatments at the same hour using ANOVA (*P < 0.05).

**Figure 2** displays the trend of fruit-zone air temperature during the warmest part of the day, demonstrating a reduction in WS+FOG vines ranging from 1.8 to 3.6°C in both seasons, compared to untreated vines. As described above, when the canopy air temperatures exceeded 35°C, the system was capable of reducing the air temperature around the clusters.

In detail for 2022, the system performed 4 cooling cycles during DOY 216 and only 2 cycles for the DOY 222 (**Figures 2A, B**). In contrast, the system’s response to the warm 2023 was different. In this case, 7 and 16 5-min misting water distribution for DOYs 228 and 233 were recorded (**Figures 2C, D**). In the same year, there is also evidence of a thermal rise in the afternoon starting at 15:00 h and a prompt response of the system to bring the temperature back below the thermal limit.

The fruit-zone cooling system was also effective on preserving leaves efficiency, shielding them from the adverse effects of high temperatures and maintaining their fundamental physiological functions. Specifically, the measurements, in two representative days during the trial for each year, showed that when the system was activated, WS+FOG exhibited higher values of Pn and gs compared to WS alone (**Tables 1**, **2**), achieving good levels, despite in some cases significantly lower as compared to the irrigated control (WW). Moreover, especially during the first year of the trial, activating the misting system resulted in a positive response in terms of gas exchange for WS+FOG, almost in line with irrigated control (WW). The WS vines instead exhibited a 50% reduction in net photosynthesis in both varieties, accompanied by a significant decline in stomatal conductance (**Tables 1**, **2**). The contrasting behavior is highlighted by water use efficiency (WUEi). Specifically, WUEi demonstrated a notable rise in vines subjected to water stress (WS) compared to well-watered (WW) and WS combined with fog (WS+FOG) treatments in both cultivars. Particularly noteworthy is the doubling of WUEi under WS conditions in the Sangiovese cultivar, as illustrated in **Table 1**. Regarding Ci, the substomatal concentration of CO_2_, it consistently decreased under WS conditions compared to the other treatments in both varieties. Checking the response of the varieties it becomes clear that after nearly 10 days of water restriction (DOY 222, **Table 1**), gas exchanges did not vary among Sangiovese and Montepulciano. However, this pattern changed after 20 days (DOY 235, **Table 2**) when WW-Sangiovese, exhibited lower values of Pn and gs compared to WW-Montepulciano, and the same trend was confirmed for the WS+FOG treatment (**Table 2**). The values of the WS vines remained significantly lower than the other treatments for both varieties under investigation, highlighting the strong multiple and synergistic effect of heat and water stress in vine physiology. This trend resulted in improved water use efficiency for water-stressed (WS) conditions (p=0.07) compared to the other treatments. Additionally, on DOY 235, the Sangiovese cultivar demonstrated significantly higher WUEi than Montepulciano (**Table 2**). Notably, on this day of physiological measurements, the substomatal concentration of CO_2_ was significantly lower compared to the other treatments in both varieties (**Table 2**).

**Table 1:** Effect of mist cooling system on net photosynthesis (Pn, µmolm-2s-1), stomatal conductance (gs, molm-2s-1), intrinsic water-use efficiency (WUEi, µmolCO_2_mol-1H_2_O), substomatal CO_2_ concentration (Ci, µmolCO_2_mol-1air), stem water potential (Ψstem, Bar) and berry temperatures (Tberry, °C) recorded at midday period on potted Sangiovese and Montepulciano vines.

Treatment	Sangiovese			Montepulciano			Significance		
	WW	WS	WS + FOG	WW	WS	WS + FOG	var	trt	interactions
**Pn**	19.4 a	6.7 c	15.5 b	21.8 a	10.9 c	17.0 b	*ns*	***	*ns*
**g_s_**	0.217 a	0.039 c	0.191 b	0.242 a	0.082 c	0.190 b	*ns*	***	*ns*
**WUEi**	89 b	170 a	82 b	90 b	139 a	94 b	*ns*	***	*ns*
**Ci**	223 a	106 b	236 a	219 a	152 b	216 a	*ns*	***	*ns*
**Ψ_stem_**	-4.8 a	-8.0 c	-7.2 b	-4.1 a	-10.0 c	-6.4 b	*ns*	***	*ns*
**T_berry_**	34.2 b	39.2 a	32.8 c	33.7 b	36.2 a	32.6 c	*ns*	*ns*	*var x trt* *

Year 2022, August 10th (DOY 222). Means within rows designated by different letters indicate significant differences among treatments. In the presence of significant variations among multiple fixed factors and the occurrence of interactions, differences between varieties (var) and treatments (trt) are denoted by asterisks according to ANOVA test (*, P < 0.05; **, P < 0.01; ***, P < 0.001, ns, not significant). Pairwise separation was performed by Tukey test.

**Table 2:** Effect of mist cooling system on net photosynthesis (Pn, µmolm-2s-1), stomatal conductance (gs, molm-2s-1), intrinsic water-use efficiency (WUEi, µmolCO_2_mol-1H_2_O), substomatal CO_2_ concentration (Ci, µmolCO_2_mol-1air), stem water potential (Ψstem, Bar) and berry temperatures (Tberry, °C) recorded at midday period on potted Sangiovese and Montepulciano vines.

Treatment	Sangiovese			Montepulciano			Significance		
	WW	WS	WS + FOG	WW	WS	WS + FOG	var	trt	interactions
**Pn**	15.8 a	7.5 c	9.1 b	19.6 a	7.8 c	14.7 b	*ns*	*	*ns*
**g_s_**	0.141 a	0.037 b	0.100 a	0.290 a	0.036b	0.223 a	*ns*	*ns*	*var x trt* **
**WUEi**	89	172	81	90	133	89	*	*ns*	*ns*
**Ci**	239 a	61 b	236 a	284 a	30 b	292 a	*ns*	***	*ns*
**Ψ_stem_**	-5.8 a	-16.5 b	-7.0 a	-6.5 a	-16.5 b	-7.9 a	*ns*	*	*ns*
**T_berry_**	34.1 b	36.6 a	29.5 d	34.6 b	35.4 a	31.3 c	*ns*	*ns*	*var x trt* *

Year 2022, August 23th (DOY 235). Means within rows designated by different letters indicate significant differences among treatments. In the presence of significant variations among multiple fixed factors and the occurrence of interactions, differences between varieties (var) and treatments (trt) are denoted by asterisks according to ANOVA test (*, P < 0.05; **, P < 0.01; ***, P < 0.001, ns, not significant). Pairwise separation was performed by Tukey test.

**Table 1** and **Table 2** also show the effect of the treatments on the vine water status (Ψstem) and the temperature of the berries. The value of stem water potential follows the same trend as stomatal conductance: the least negative values are recorded in WW treatments, the most negative in WS theses, while WS+FOG reaches an intermediate value, indicating a significant effect of misting on this parameter. The thermal response of berries to treatments differs. Specifically, WS+FOG reduced the berry temperature below the thermal threshold of 35°C on both the two survey days. This thermal difference averaged 6-7°C for Sangiovese and 4°C for Montepulciano compared to WS. WW took an intermediate value for the two varieties but it was still significantly lower than WS (**Tables 1**, **2**).

The same variables investigated in 2022 were evaluated in two representative days (DOY 219 and 223) of 2023, picked within a period characterized by severe heatwaves and very low rainfall (**Tables 3** **4**). In contrast to the previous year, one week after setting irrigation restriction, net assimilation did not appear to differ between treatments. Instead, a significant difference between the compared varieties was recorded, with Montepulciano showing better photosynthetic efficiency than Sangiovese (**Table 3**). Specifically, the reduction in irrigation (-50% of water lost through transpiration in WS and WS+FOG compared to WW) led to a notable decrease in both stomatal conductance and stem water potential. Simultaneously, there was an observed increase in water use efficiency and a decrease in substomatal CO_2_ concentration (**Table 3**). No significant differences were found between treatments in berry temperature because - at the time of the measurement - the system had not been activated since the air temperature was below the threshold, 35° C (**Table 3**; **Figure 1B**). Vine responses changed with the increasing number of days of water stress (DOY 223), as highlighted in **Table 4**. In detail, the occurrence of multiple summer stress factors (thermal and radiative) associated with water scarcity, negatively affects the efficiency of photosystems and stomatal activity in WS and WS+FOG. Both treatments indeed exhibit near-zero assimilation and stomatal conductance values below 0.05 mol m^-2^ s^-1^. Moreover, there was no discernible differences in water use efficiency. Surprisingly, the Ci for these treatments under irrigation restriction exceeded the values observed in well-watered conditions (WW) for both varieties (**Table 4**). In particular, the vines subjected to water restriction exhibited a decrease in stem water potential, reaching values almost doubled compared to the irrigated control (**Table 4**). In terms of berry temperature, the activation of the misting system has allowed lowering the temperature of the berries by about 3°C compared to WS and 1°C compared to WW for both cultivars (**Table 4**).

**Table 3:** Effect of mist cooling system on net photosynthesis (Pn, µmolm-2s-1), stomatal conductance (gs, molm-2s-1), intrinsic water-use efficiency (WUEi, µmolCO_2_mol-1H_2_O), substomatal CO_2_ concentration (Ci, µmolCO_2_mol-1air), stem water potential (Ψstem, Bar) and berry temperatures (Tberry, °C) recorded at midday period on potted Sangiovese and Montepulciano vines.

Treatment	Sangiovese			Montepulciano			Significance		
	WW	WS	WS + FOG	WW	WS	WS + FOG	var	trt	interactions
**Pn**	10.5 b	7.8 b	8.8 b	10.7 a	10.1a	10.5 a	*	*ns*	*ns*
**g_s_**	0.202 a	0.101 b	0.127 b	0.207 a	0.136 b	0.162 b	*ns*	***	*ns*
**WUEi**	51 b	77 a	70 a	52 b	74 a	64 a	*ns*	***	*ns*
**Ci**	288 a	247 b	266 b	295 a	249 b	260 b	*ns*	***	*ns*
**Ψ_stem_**	-6.2 a	-10.0 b	-8.3 b	-4.1 a	-9.5 b	-8.1 b	*ns*	*	*ns*
**T_berry_**	28.8	28.3	28.0	27.0	27.0	26.5	*ns*	*ns*	*ns*

Year 2023, August 7th (DOY 219). Means within rows designated by different letters indicate significant differences among treatments. In the presence of significant variations among multiple fixed factors and the occurrence of interactions, differences between varieties (var) and treatments (trt) are denoted by asterisks according to ANOVA test (*, P < 0.05; **, P < 0.01; ***, P < 0.001, ns, not significant). Pairwise separation was performed by Tukey test.

**Table 4:** Effect of mist cooling system on net photosynthesis (Pn, µmolm-2s-1), stomatal conductance (gs, molm-2s-1), intrinsic water-use efficiency (WUEi, µmolCO_2_mol-1H_2_O), substomatal CO_2_ concentration (Ci, µmolCO_2_mol-1air), stem water potential (Ψstem, Bar) and berry temperatures (Tberry, °C) recorded at midday period on potted Sangiovese and Montepulciano vines.

Treatment	Sangiovese			Montepulciano			Significance		
	WW	WS	WS + FOG	WW	WS	WS + FOG	var	trt	interactions
**Pn**	11.8 a	1.0 b	1.5 b	12.4 a	1.5 b	3.7 b	***	***	*ns*
**g_s_**	0.230 a	0.027 b	0.049 b	0.203 a	0.022 b	0.047 b	*ns*	***	*ns*
**WUEi**	60	40	61	62	72	75	*ns*	*ns*	*ns*
**Ci**	275 b	461 a	322 b	272 b	343 a	294 b	*ns*	***	*ns*
**Ψ_stem_**	-10.3 a	-18.5 c	-14.1 b	-9.6 a	-18.6 c	-12.3 b	*ns*	**	*ns*
**T_berry_**	34.8 b	36.4 a	33.7 c	34.9 b	37.3 a	33.7 c	*ns*	***	*ns*

Year 2023, August 11th (DOY 223). Means within rows designated by different letters indicate significant differences among treatments. In the presence of significant variations among multiple fixed factors and the occurrence of interactions, differences between varieties (var) and treatments (trt) are denoted by asterisks according to ANOVA test (*, P < 0.05; **, P < 0.01; ***, P < 0.001, ns, not significant). Pairwise separation was performed by Tukey test.

**Figure 3:**
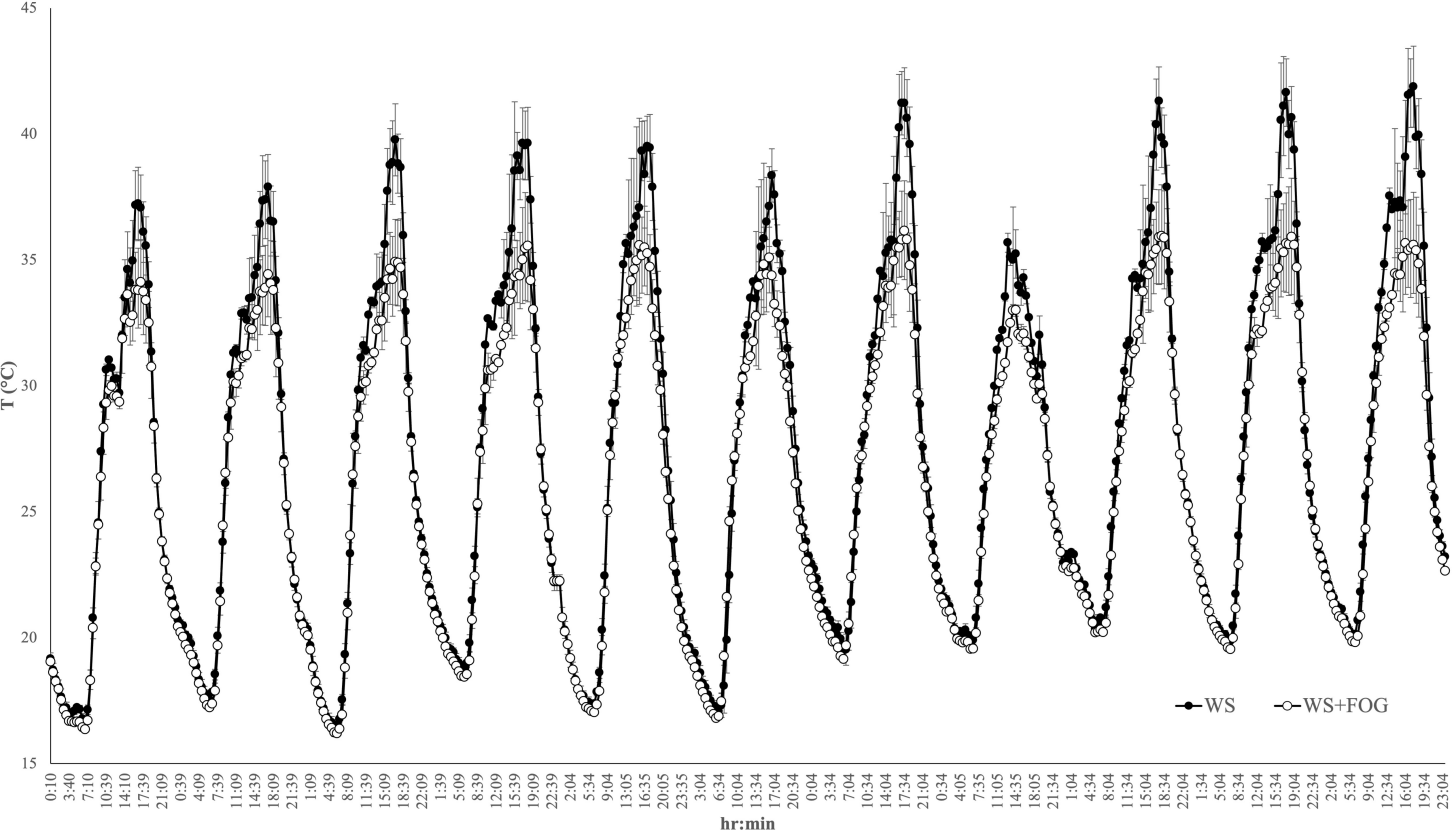
Trend of WS and WS+FOG berry temperatures during eleven days of August 2023 (DOY 222-DOY 232). Each data point represents the average of three thermocouples inserted into the berries facing east, west, and in the middle of the vegetative wall. Error bars indicate the standard error.

To gain a deeper insight into the impact of the fruit-zone cooling system on cluster temperature, in 2023, thermocouples were placed on various berries associated with both WS and WS+FOG theses at different points within the canopy. **Figure 3** illustrates the temperature fluctuations over eleven-days in August, representative of the heatwaves period. Notably, it is evident that WS exhibited a distinct temperature trend compared to WS+FOG from solar noon to 19:00 hours. This divergence reached its peak on DOY 232, with a temperature difference of 6°C between the two treatments. It is noteworthy that berries subjected to misting consistently maintain temperatures below the critical limit of 35°C, as initially identified through a timely infrared thermometer survey (**Figure 3**).

### 3.3 Yield components and berry composition

Despite a uniform number of clusters per vine, significant differences between treatments were reported on yield components (**Table 5**). Throughout the two-year trial period, a substantial 15% decrease of yield was observed in the WS thesis compared to WW in both varieties. This decline can be primarily attributed to the reduction of bunch weight, with the most pronounced effect evident in 2023 in both varieties (**Table 5**). The misting system emerged as a pivotal factor influencing yield. Specifically, WS+FOG demonstrated heavier clusters, aligning closely with the irrigated control, resulting in a significant yield increase compared to WS (**Table 5**). Conversely, when considering berry mass, a significant difference is observed between the two vintages, with 2023 exhibiting greater weights than 2022. However, the impact of treatments on the two varieties is less straightforward. Notably, WW consistently produces the largest berries, while WS yields the smallest. WS+FOG aligns with WW in berry size, particularly for Montepulciano. Conversely, it appears that the misting system does not significantly influence berry mass for Sangiovese.

**Table 5:** Yield attributes and berry composition parameters measured at harvest in Sangiovese and Montepulciano vines over 2022-2023 seasons (TSS, total soluble solids; TA, total acidity).

Treatment	Sangiovese			Montepulciano			Significance			
	WW	WS	WS+FOG	WW	WS	WS+FOG	year	var	trt	interactions
Yield (kg vine^-1^)	1.47 a	1.27 b	1.49 a	1.56 a	1.37 b	1.64 a	*ns*	*ns*	*	*ns*
2022	1.39	1.42	1.60	1.48	1.28	1.52				
2023	1.54	1.12	1.37	1.63	1.45	1.76				
Cluster weight (g)	128.7 a	109.8 b	125.1 a	123.3 a	107.3 b	126.3 a	***	*ns*	*	*ns*
2022	109.8	108.5	117.5	110.0	103.2	113.7				
2023	147.7	110.8	132.7	136.7	111.3	138.9				
Berry mass (g)	1.74 a	1.68 b	1.66 b	1.98 a	1.51 b	1.91 a	***	*ns*	*ns*	*var x trt* *
2022	1.55	1.54	1.44	1.72	1.30	1.60				
2023	1.93	1.83	1.88	2.24	1.73	2.23				
TSS (°Brix)	22.2	22.4	22.5	23.5	22.0	22.7	*ns*	*ns*	*ns*	*year x var x trt* *
2022	20.9	21.8	21.9	23.6.	23.9	23.0				
2023	23.5	23.0	23.0	23.4	20.2	22.3				
pH	3.56 b	3.60 ab	3.67 a	3.47 a	3.44 a	3.49 a	***	*ns*	*ns*	*var x trt* *
2022	3.58	3.64	3.79	3.48	3.49	3.54				
2023	3.47	3.54	3.55	3.46	3.39	3.44				
TA (g L^-1^)	6.77	6.89	6.20	6.13	6.54	6.04	***	**	*ns*	*ns*
2022	6.59	6.29	5.32	5.57	5.95	5.15				
2023	6.94	7.48	7.07	6.68	7.52	6.93				
Total anthocyanins (mg kg^-1^)	638.8 b	580.5 c	759.2 a	1420.3 b	1308.7 c	1547.4 a	*	*	*	*ns*
2022	519.9	462.5	735.4	1353.6	1298.3	1578.8				
2023	847.7	698.4	783.0	1487.0	1319.0	1516.0				

Means within rows designated by different letters indicate significant differences among treatments (n=5). In the presence of significant variations among multiple fixed factors and the occurrence of interactions, differences between varieties (var), treatments (trt), and years (year) are denoted by asterisks according to ANOVA test (*, P < 0.05; **, P < 0.01; ***, P < 0.001, ns, not significant). The table presents pairwise comparisons conducted with Tukey’s test for further insights into the observed distinctions.

**Figure 4:**
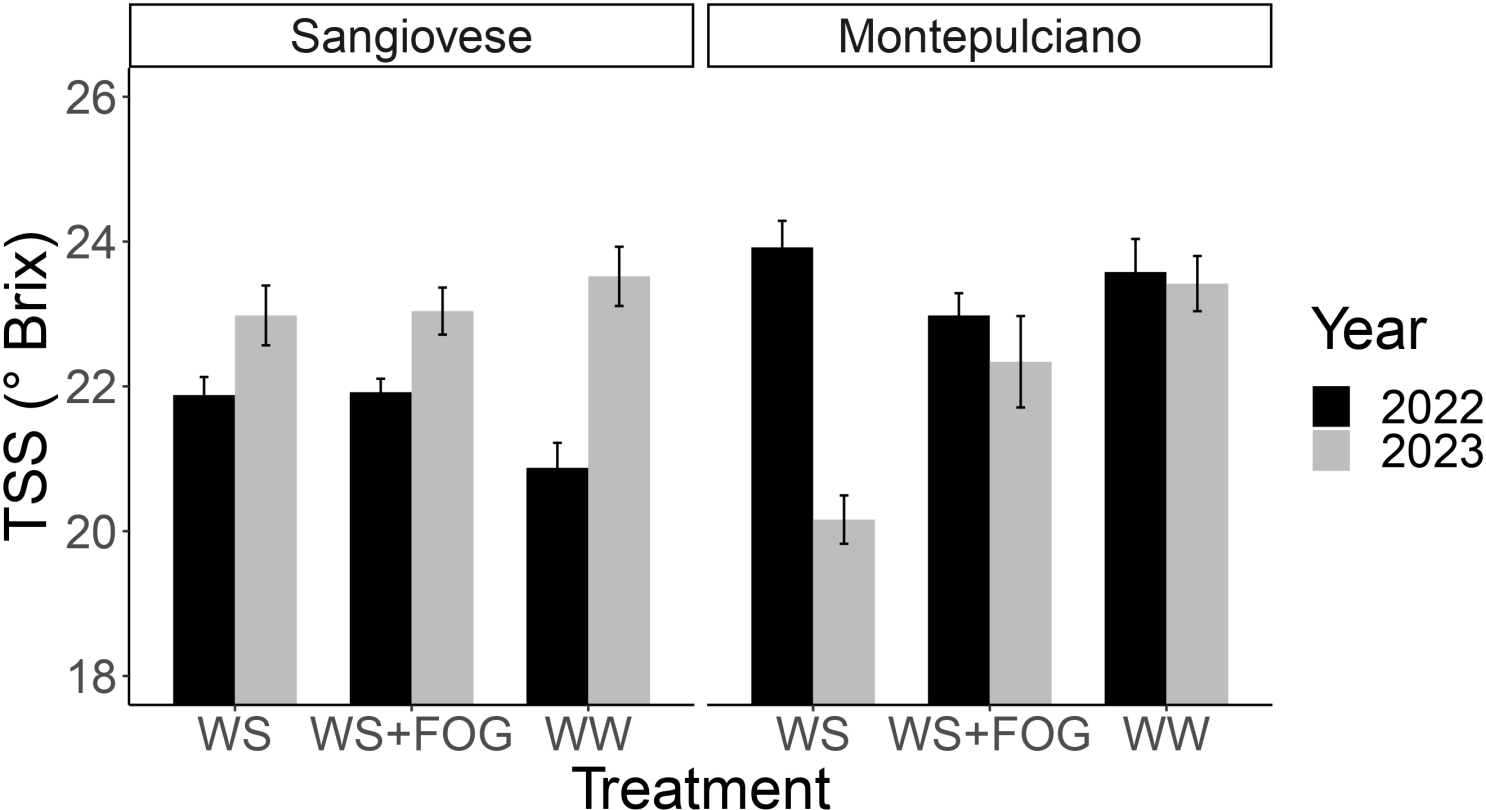
Bar chart illustrating the sugar concentration (TSS) at harvest for the years 2022 and 2023. The interaction among different factors - varieties, years and treatments - is evident. The two bar charts correspond to the two varieties under examination: Sangiovese and Montepulciano (n=5, error bars depict the standard error).

A year x variety x treatment interaction was observed concerning soluble solids at harvest (**Table 5**). To provide a clearer understanding of the results, the values for each treatment in the two test years are presented in **Figure 4**. The analysis of the bar chart offers several insights: in 2022, Montepulciano exhibited, on average, a higher Total Soluble Solids (TSS) value than Sangiovese, with no discernible differences between treatments. In 2023, a year marked by exceptionally high pre-harvest temperatures, notable differences in the response of the WS treatment for the two varieties became apparent. Specifically, the accumulation of TSS in Montepulciano was significantly lower than in Sangiovese, by approximately 2°Brix (**Table 5**). Interestingly, this pattern did not extend to WS+FOG, where equivalent sugar levels were reinstated for each variety, as observed in WW (**Figure 4**).

With regard to the pH and the acidity profile of the grapes, it is widely recognized that various factors play a role, including the vegetative development of the plants, climatic conditions before the veraison stage, berry volume and abiotic factors such as temperature. A thorough examination of the total acidity values recorded in harvested grapes reveals a notable distinction between the two years: 2022 exhibited a higher pH and consequently lower acidity level compared to 2023 (**Table 5**). Additionally, significant variations among grape varieties are evident, with Montepulciano displaying lower organic acid content than Sangiovese. However, treatments do not appear to exert any discernible influence on the berry acidity levels (**Table 5**). Microclimate conditions could have also a great impact on phenolic accumulation and composition during berry ripening (**Table 5**). Concerning the concentration of total anthocyanins, a significant enhancement was observed in the WS+FOG treatment compared to both WS and WW. Despite anthocyanin accumulation capacity is different between the two varieties due to genetic factors, both of them exhibited a positive response to the application of misting in the cluster zone. Specifically, WS showed a 16 to 22 percent reduction of anthocyanin concentration compared to WS+FOG in Montepulciano and Sangiovese, respectively (**Table 5**).

## 4 Discussion

In light of the proven capacity of water misting to lower the surface temperature of vine organs (Howell et al., 1971; Kliewer and Schultz, 1973), the aim of the two-year trial was to investigate the impact of an innovative fruit-zone cooling system on two grape varieties: Sangiovese and Montepulciano. As well known, these varieties are recognized for exhibiting distinct responses under conditions of both limited and unlimited water availability, particularly in the context of climate change (Palliotti et al., 2011; Palliotti et al., 2014).

The system, originally configured to activate on the base of predetermined thresholds of vapor pressure deficit (VPD), was specifically adapted in 2022 and 2023 seasons to respond exclusively to thermal limits (T≥35°C). This decision was thoughtfully made, taking into consideration the climatic conditions of the vineyard (Bologna, Italy) which are distinguished by almost constant humidity during summer, coupled with elevated temperatures (Teslić et al., 2019). In detail, the Mediterranean basin has been facing increasingly harsh years, consequently, vines are exposed to intense solar radiation, elevating the risk of excessive temperature rise around the fruit-zone (Poni et al., 1994; Valentini et al., 2022). This can result in reduced yields and a decline of grape quality, including anthocyanin accumulation (de Orduna, 2010) which may be exacerbated when coupled with a substantial reduction of soil water content (Carvalho et al., 2016).

Given these challenges, the misting system was implemented at the experimental station of the University of Bologna to evaluate its impact on potted vines subjected to water restriction starting from veraison (WS+FOG) compared to both water-stressed (WS) vines and irrigated control (WW). Various parameters were meticulously assessed during this phase, encompassing not only the temperature of the fruit-zone and clusters but also gas-exchange, yield attributes and berry composition.

The innovative misting system effectively cools both the air surrounding the clusters and the clusters themselves, using minimal amount of water. Specifically, in the warm conditions of the 2022 season, the system was employed for 7 days, distributing approximately 10 mm of water cumulatively by the trial’s conclusion. However, during the challenging 2023 season, elevated temperatures were recorded, leading to more frequent activations of the fruit zone cooling system and a greater volume of water distributed, especially in the latter part of August. Despite the elevated temperatures, the system performed effectively by not only cooling the air near the clusters but also maintaining the temperature of the clusters below the 35°C thermal threshold during heatwaves. These results are in line with those obtained by other similar devices designed to preserve water resources (Greer and Weedon, 2014; Caravia et al., 2016; Bianchi et al., 2023). In detail, the fruit-zone cooling system showed some innovative features: it minimized water usage through real-time thermo-hygrometric data readings (approximately 1 mm of water per hour), operated automatically and employed an advanced Wireless Sensor Network (WSN) for precise misting. In this way, two fundamental requirements for water resource conservation were met: the quantity of water used and the distribution method.

The fruit-zone cooling system demonstrated significant effects on leaf photosynthetic efficiency, alleviating the adverse impacts of high temperatures and preserving crucial physiological functions. As indicated by Zheng et al. (2021), the reduction of temperature during berry ripening positively influenced leaf gas exchanges, such as net photosynthesis. Consequently, activating the misting system to water-stressed vines yielded higher levels of photosynthesis and stomatal conductance compared to vines experiencing water stress alone. We hypothesized that the system strengthened this response by directly influencing the vapor pressure deficit (VPD) in the treated fruit zone, as reported in a previous study where the VPD of misted vines was halved compared to untreated control (Pagay et al., 2018). In our conditions, air temperature control through the fruit-zone cooling system was the main factor determining the reduction of vapor pressure deficit, with humidity generally remaining constant. The positive response of gas-exchange was particularly pronounced in the first year, almost aligning WS+FOG with the irrigated control (WW), while WS exhibited a 50 percent reduction in net photosynthesis accompanied by a decline of stomatal conductance. This phenomenon is associated with the varying microclimatic conditions within the canopy and the increased evaporative demand, impacting stomatal response in the short term. As widely recognized, stomata typically close to minimize water loss, resulting in decreased CO_2_ assimilation (Flexas et al., 2002; Escalona et al., 2003). This trend was observed in WS vines, which showed an inclination towards heightened water use efficiency. Furthermore, during the 2022 season, this trend seemed correlated with the Sangiovese variety, exhibiting higher WUEi compared to Montepulciano. However, as water stress conditions intensified, exacerbated by exceptionally high temperatures experienced during the 2023 season, a notable decrease in both photosynthesis and stomatal conductance was evident (Gómez-del-Campo et al., 2002; Cifre et al., 2005; de Souza et al., 2005; Chaves et al., 2007). While mild water stress typically depresses grapevine photosynthesis predominantly through stomatal closure (as evidenced by the improved WUEi), more severe water stress scenarios are known to involve non-stomatal inhibition of photosynthesis (Chaves, 1991; Flexas et al., 2002). During this phase, a decrease in WUEi and an increase in Ci indicate the dominance of non-stomatal limitations on photosynthesis (Cifre et al., 2005).

Additionally, the Montepulciano vines confirmed the greater physiological and productive performance compared to those of Sangiovese, under conditions of adequate irrigation (Palliotti et al., 2011). Specifically, during the 2022 season, after 20 days of water restriction, it was observed that stomatal conductance values of WS+FOG and WW were higher in Montepulciano while the response profile to WS was similar for both varieties. These results align with findings reported by Greer and Weedon (2014), emphasizing the impact of high temperatures on the Semillon variety and their depressive effect on gas-exchange. The enhanced performance of Montepulciano in comparison to Sangiovese was also evident in 2023. However, during this year, distinguishing differences between treatments subjected to water restriction became challenging due to the heightened presence of multiple stresses affecting both the WS and WS+FOG treatments (Martínez-Lüscher et al., 2020).

It is widely acknowledged that grapevines are generally classified as anisohydric (Schultz, 2003), and they typically exercise precise control over stomatal opening that adapt to preserve their water status under drought conditions. This regulatory mechanism leads to a reduction of the leaf and stem water potential under conditions of water stress (Tardieu and Simonneau, 1998). Across the two years of the trial, Ψ_stem_ exhibited a trend similar to that of stomatal conductance, with the least negative values in WW, the most negative in WS, and an intermediate value in WS+FOG, underscoring the significant impact of misting on canopy microclimate. As reported in literature, Ψ_stem_ is considered a reliable index of water status in *Vitis vinifera* L., and its values are the combination of different factors, such as VPD, soil water availability, stomatal regulation and plant hydraulic conductivity (Patakas et al., 2005). Manipulating the microclimate in the cluster zone sets off a chain reaction, influencing the plant’s gas exchange processes and vine water status without directly supplying water to the soil. In our experiment, the pots were covered with plastic foil, and the water distributed by the system did not reach the ground. These findings align with research by Pagay et al. (2018) and studies on the Hydrocooling technique (Greer and Weedon, 2014), where fine mist, in direct contact with the vegetative surface, evaporates rapidly without reaching the soil. In our case, the berry temperature seems to show significant differences over time between varieties and treatments. As observed by Pagay et al. (2018), treated leaves dry faster during heatwaves (in less than one minute), unlike clusters due to their different thermal masses.

Reducing cluster temperature proved advantageous for both yield and berry composition. Notably, non-misted vines experienced a significant decrease in yield attributed to the reduction of cluster weight. This decrease was linked to an elevated occurrence of dehydration and/or necrosis phenomena, directly impacting berry mass, which are common in the ongoing climate change scenario (Bonada and Sadras, 2015; Pastore et al., 2013; Caravia et al., 2016). Grapevines typically exhibit lower sensitivity to water stress during reproductive growth compared to vegetative growth (Korkutal et al., 2019; Williams and Matthews, 1990). However, this response may vary under climate change scenario. For instance, in severe water stress condition, bunch weight diminishes with a temperature increase of 4°C (Kizildeniz et al., 2015). This situation was also observed over the 2-year trial when the temperature gap between WS and WS+FOG reached peaks of 6°C. Additional research has highlighted that when temperatures peak at 40°C, impediments in berry growth and responses in terms of sugar accumulation occur (Greer and Weston, 2010). Specifically, Semillon berries at the veraison stage exhibited high sensitivity to heat, leading to a significantly restricted growth phase and disrupted sugar accumulation. Simultaneously, there was a sustained decline of leaf photosynthesis caused by heat, impacting the carbon supply available to meet the demands of the berries.

In our trial, soluble solids (TSS) exhibited varietal and vintage differences. In 2023, when the highest temperatures were recorded, WS-Montepulciano exhibited lower TSS, but WS+FOG vines restored similar sugar levels to WW-ones. This result is mostly associated with high temperatures during veraison and mid-ripening that affect the formation and translocation of sugars, resulting in a significant reduction (Greer and Weston, 2010). The process of sucrose loading into the grape berry involves multiple sugar transporters and sucrose metabolic enzymes. High temperatures have been found to inhibit these metabolic processes (Agasse et al., 2009). Moreover, elevated temperatures can lead to the inactivation of specific genes in the berries that play a role in the metabolism of berry ripening (Pillet et al., 2012). Montepulciano is particularly sensitive to the interaction between water scarcity and high temperatures (Dal Santo et al., 2016; Tombesi et al., 2016). Furthermore, significant differences in the response of the two varieties to the water stress imposed during veraison are evident. It is clear that what occurred in WS-Montepulciano did not occur in WS-Sangiovese, which did not show a significant decrease of soluble solids at harvest. This is attributed to the higher accumulation of ABA in the leaves of Montepulciano affecting the carbon assimilation at the whole canopy level (Soar et al., 2006; Dal Santo et al., 2016) and the excellent recovery capacity of Sangiovese during rehydration (Palliotti et al., 2014). If it is well known that the effect of water stress is more significant when imposed before veraison (Keller, 2005), this study demonstrates that when water stress acts synergistically with thermal stress, the response of sugar accumulation can vary depending on the grapevine genetics. Additionally, it suggests that fruit zone cooling can prevent a maturation halt. Furthermore, in a vintage characterized by severe water and heat stress, we can confirm that Sangiovese manages to maintain higher performance and ensure the complete ripening of the berries.

Acidity levels displayed variability across years, with no discernible impact from the treatments. As discussed by Sadras and Moran (2012), the influence of high temperatures on titratable acidity and pH is intricate, influenced by factors such as cultivar, season and their interactions. In detail, the analysis of phenotypic plasticity unveiled distinct responses for Chardonnay, Cabernet Franc, Semillon and Shiraz, the latter showcasing a notable lack of plasticity by maintaining stable acidity and pH under diverse conditions. This challenges the prevailing notion that larger berry would exhibit dilution effects in both organic acids and sugars. Our findings suggest that grapes of varying masses attained similar technological maturities. These outcomes align with those observed in White Riesling by Kliewer (1973), referencing data collected in 1971 and those obtained in Cabernet Sauvignon (Caravia et al., 2017). Thus, it seems that in all the different varieties, cooling the vines by sprinkling water has an important effect on berry growth, but little or no effect on the berry ripening process (Aljibury et al., 1975; Caravia et al., 2016).

On the contrary, the fruit-zone cooling system demonstrated promising outcomes concerning the accumulation of polyphenols such as anthocyanins. The treatment effect was significant in both years, indicating that under both multiple stress (WS) and only heat stress (WW) conditions, water spraying in the bunch zone leads to an enhancement of grape color. Our findings were primarily attributed to the cooling effect, fostering anthocyanin biosynthesis stimulation and reducing anthocyanin degradation phenomena (Allegro et al., 2021). Pastore et al. (2017) highlighted that under moderate temperature conditions, the interaction of light and temperature is synergistic, while in the presence of heatwaves, an antagonistic effect emerges. The mechanism involves the upregulation of oxidative enzymes, such as peroxidases (PODs), and a diminished efficiency in the expression of genes related to biosynthesis (Movahed et al., 2016). The adverse impacts of elevated temperatures may be exacerbated when coupled with a substantial reduction of soil water content (Carvalho et al., 2016). In conclusion, if irrigating before an extreme heat event can increase leaf transpiration, generating a cooling effect in the fruit zone similar to that of overhead irrigation with sprinklers (Kliewer and Schultz, 1973; Martínez-Lüscher et al., 2020), it is demonstrated here that the effect of a misting system dedicated to the cluster zone has a direct impact on the accumulation of anthocyanins, reducing their degradation.

## 5 Conclusions

The fruit-zone cooling system, evaluated at the University of Bologna, effectively mitigated multiple summer stresses on Sangiovese and Montepulciano vines during the 2022 and 2023 seasons marked by elevated temperatures. Specifically, the system succeeded in lowering both fruit-zone and berry temperatures, thereby enhancing canopy gas exchanges, yield production and the berry composition. In particular, the system showed a significant effect on the accumulation of anthocyanins in both Sangiovese and Montepulciano varieties. However, additional studies are necessary to assess the impact of the fruit-zone cooling system at the field level.

## Data availability statement

The raw data supporting the conclusions of this article will be made available by the authors, without undue reservation.

## Author contributions

GV: Conceptualization, Data curation, Formal Analysis, Investigation, Methodology, Visualization, Writing – original draft. GA: Data curation, Investigation, Writing – review & editing. CP: Data curation, Investigation, Writing – review & editing. DS: Data curation, Investigation, Visualization, Writing – review & editing. MN: Investigation, Software, Writing – review & editing. EM: Formal Analysis, Writing – review & editing. IF: Conceptualization, Methodology, Supervision, Writing – review & editing.
